# Supplementary material for: Unraveling the complexity of nurses’ quality of life: A Fuzzy-Set Qualitative Comparative Analysis (fsQCA) approach to the interplay of psychological, occupational, and social factors
Source: PLoS One. 2026 May 26;21(5):e0348584. doi: 10.1371/journal.pone.0348584 (PMC13210195; doi:10.1371/journal.pone.0348584)
Supplement: Attachment — Submitted filename: plos one REVIEW_DORIS HAGAN.docx [file pone.0348584.s001.docx]

"Unraveling the Complexity of Nurses' Quality of Life: A QCA Approach to the Interplay of Psychological, Occupational, and Social Factors"

Strengths:

1. Timely and Relevant Topic: The manuscript addresses a critical issue in healthcare nurses' quality of life (QOL) which has significant implications for healthcare delivery, patient outcomes, and workforce sustainability.
2. Innovative Methodology: The use of fuzzy-set Qualitative Comparative Analysis (fsQCA) is a novel approach that provides a contextual understanding of the interplay between psychological, occupational, and social factors affecting nurses' QOL.
3. Comprehensive Analysis: The study identifies specific configuration pathways for high and low QOL, offering actionable insights for targeted interventions.
4. Data Collection: The study includes a large sample size (503 nurses) from tertiary hospitals, ensuring a solid foundation for statistical analysis. The use of validated scales with high reliability (Cronbach’s α ranging from 0.81 to 0.92) strengthens the credibility of the findings.
5. Presentation of Results: The manuscript provides detailed tables and statistical analyses, including descriptive statistics, correlation analysis, hierarchical regression models, and fsQCA results, which are well-organized and easy to interpret.
6. Ethical Compliance: The study adheres to ethical standards, with approval from the Ethics Committee and informed consent obtained from participants.

Weaknesses:

1. Consider writing in full the abbreviated word ‘QCA’ in the paper title.
2. Generalizability: The use of convenience sampling and the focus on nurses from only two tertiary hospitals may limit the generalizability of the findings to other healthcare settings, such as primary care or rural hospitals.
3. Self-Reported Data: The reliance on self-reported measures may introduce response bias, such as social desirability or recall bias, which could affect the accuracy of the results.
4. Limited Contextual Factors: The study does not account for other potential contextual factors, such as organizational culture, healthcare policies, or regional differences, which may influence nurses' QOL.
5. Data Availability: The data availability statement indicates that data supporting the findings are available upon request from the corresponding author, which may limit transparency and accessibility for future research.

Recommendations:

1. Future studies should aim to include a more diverse sample of nurses from various healthcare settings to enhance the generalizability of the findings.
2. To reduce response bias, consider integrating objective measures or triangulating self-reported data with qualitative methods.
3. Include additional variables such as organizational culture, healthcare policies, and regional differences to provide a more comprehensive understanding of factors influencing nurses' QOL.
4. Consider making the data publicly available in a repository to align with PLOS ONE’s data availability policy and enhance transparency.

Conclusion: The manuscript is well-written, addresses an important topic, and employs a novel methodology to provide valuable insights into the factors influencing nurses' quality of life. While the study has some limitations, the findings offer practical implications for healthcare organizations aiming to improve nurses' well-being. Addressing the recommendations could further strengthen the manuscript and its contribution to the field.
